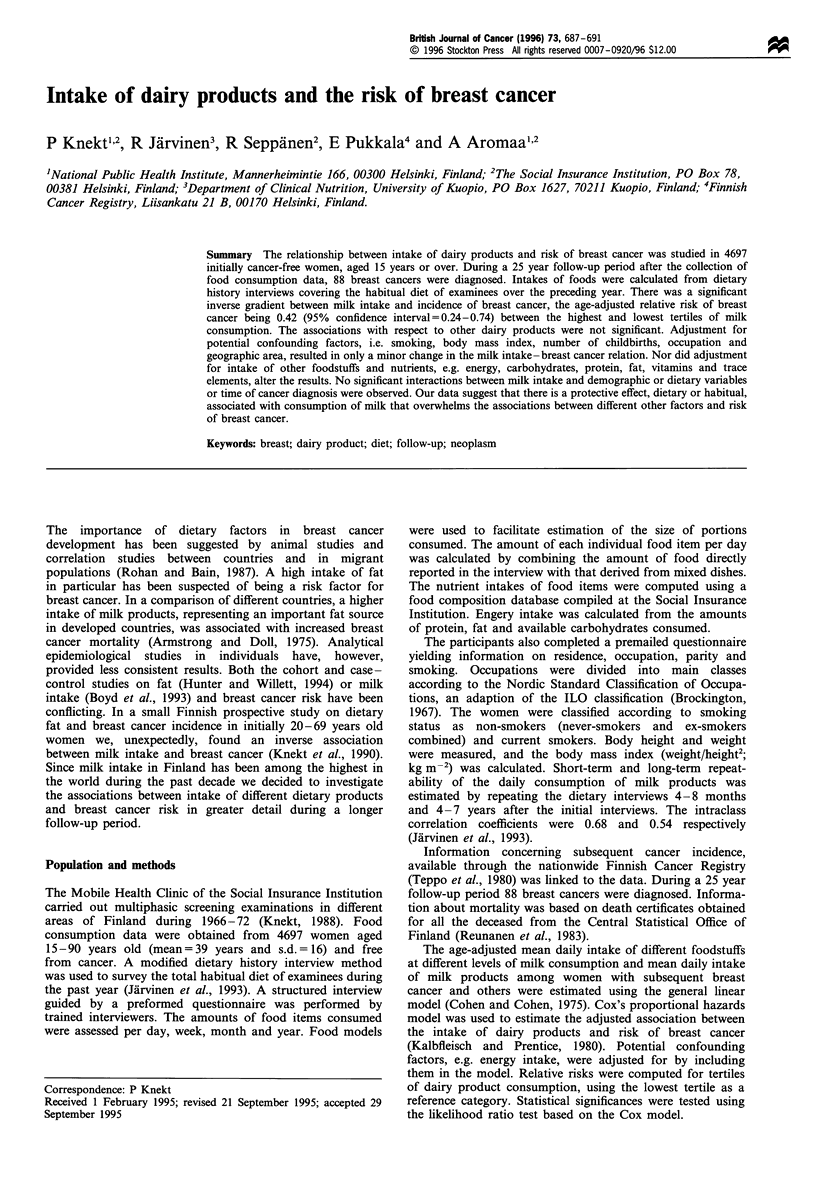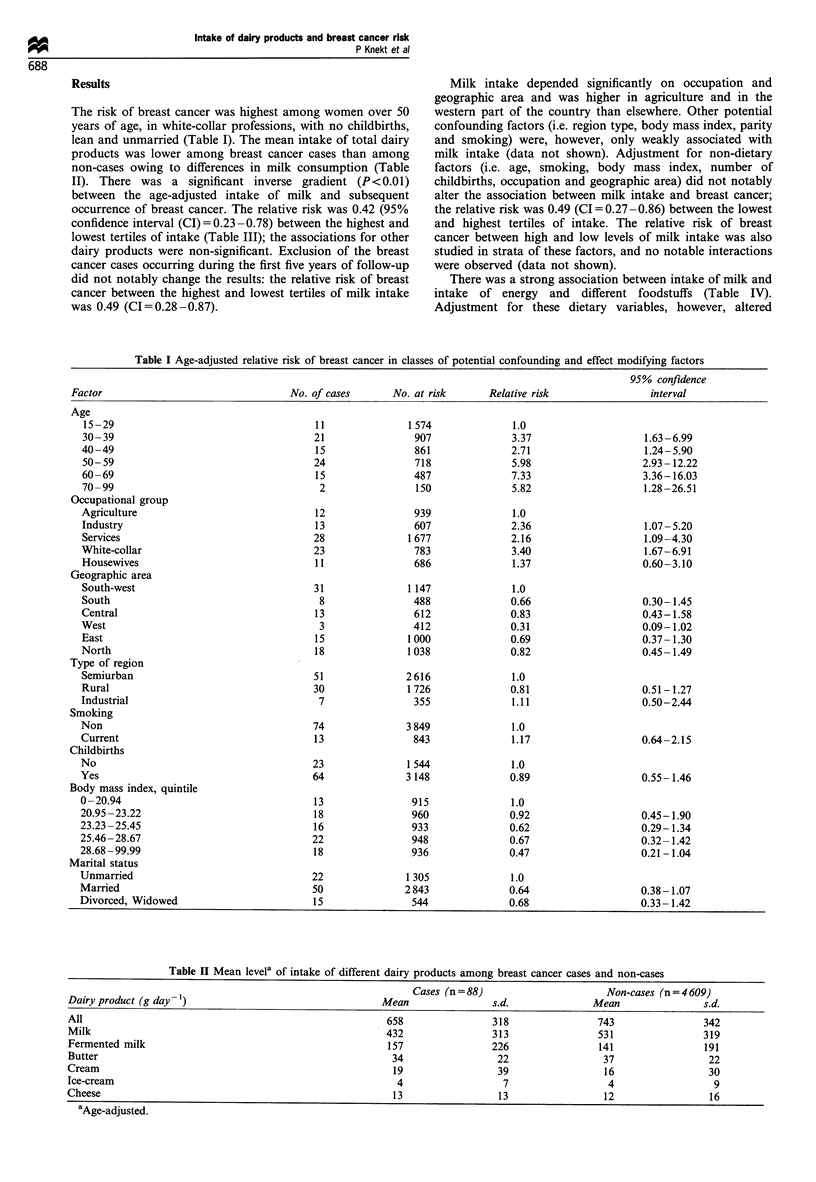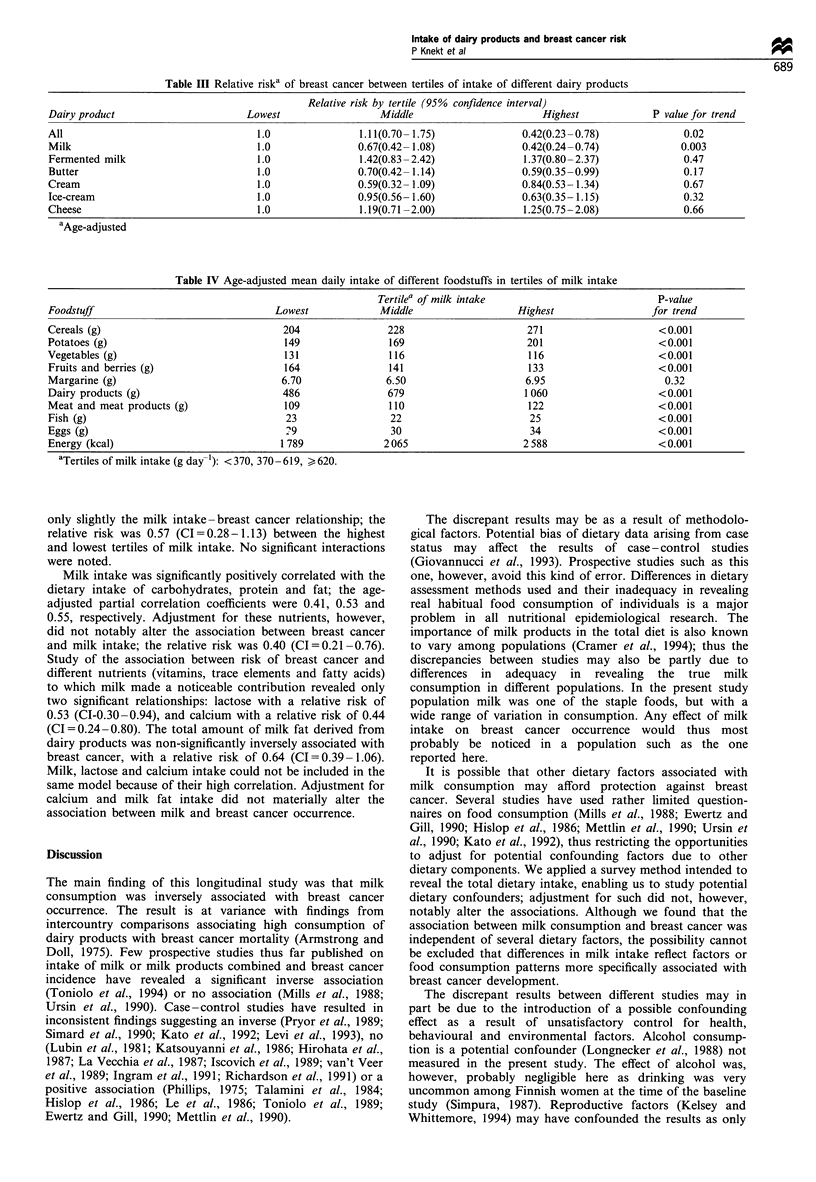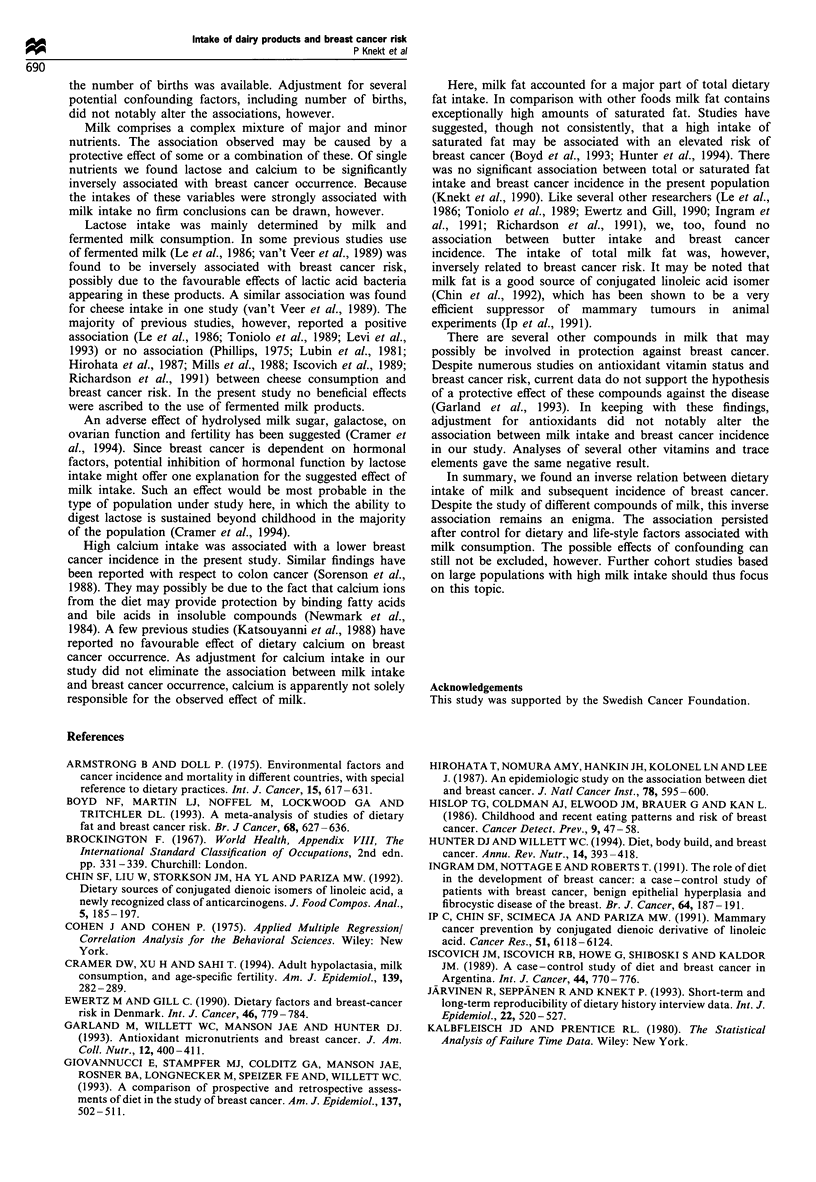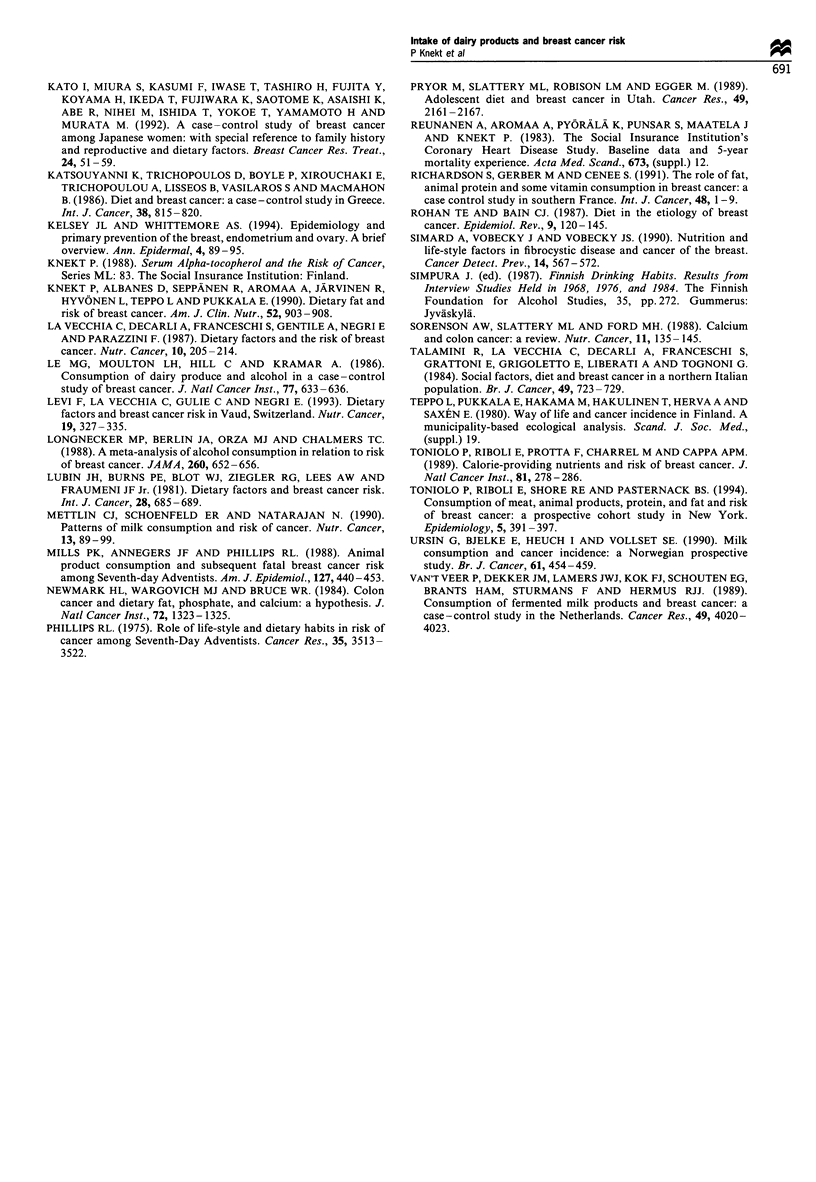# Intake of dairy products and the risk of breast cancer.

**DOI:** 10.1038/bjc.1996.119

**Published:** 1996-03

**Authors:** P. Knekt, R. Järvinen, R. Seppänen, E. Pukkala, A. Aromaa

**Affiliations:** National Public Health Insitute, Helsinki, Finland.

## Abstract

The relationship between intake of dairy products and risk of breast cancer was studied in 4697 initially cancer-free women, aged 15 years or over. During a 25 year follow-up period after the collection of food consumption data, 88 breast cancers were diagnosed. Intakes of foods were calculated from dietary history interviews covering the habitual diet of examinees over the preceding year. There was a significant inverse gradient between milk intake and incidence of breast cancer, the age-adjusted relative risk of breast cancer being 0.42 (95% confidence interval=0.24-0.74) between the highest and lowest tertiles of milk consumption. The associations with respect to other dairy products were not significant. Adjustment for potential confounding factors, i.e. smoking, body mass index, number of childbirths, occupation and geographic area, resulted in only a minor change in the milk intake-breast cancer relation. Nor did adjustment for intake of other foodstuffs and nutrients, e.g. energy, carbohydrates, protein, fat, vitamins and trace elements, alter the results. No significant interactions between milk intake and demographic or dietary variables or time of cancer diagnosis were observed. Our data suggest that there is a protective effect, dietary or habitual, associated with consumption of milk that overwhelms the associations between different other factors and risk of breast cancer.


British Journal of Cancer (1996) 73, 687-691
© 1996 Stockton Press All rights reserved 0007-0920/96 $12.00

### Intake of dairy products and the risk of breast cancer

P Knekt[1,2], R Järvinen[3], R Seppänen[2], E Pukkala[4] and A Aromaa[1,2]

[1]*National Public Health Institute, Mannerheimintie 166, 00300 Helsinki, Finland;* [2]*The Social Insurance Institution, PO Box 78, 00381 Helsinki, Finland;* [3]*Department of Clinical Nutrition, University of Kuopio, PO Box 1627, 70211 Kuopio, Finland;* [4]*Finnish Cancer Registry, Liisankatu 21 B, 00170 Helsinki, Finland.*

**Summary** The relationship between intake of dairy products and risk of breast cancer was studied in 4697 initially cancer-free women, aged 15 years or over. During a 25 year follow-up period after the collection of food consumption data, 88 breast cancers were diagnosed. Intakes of foods were calculated from dietary history interviews covering the habitual diet of examinees over the preceding year. There was a significant inverse gradient between milk intake and incidence of breast cancer, the age-adjusted relative risk of breast cancer being 0.42 (95% confidence interval = 0.24-0.74) between the highest and lowest tertiles of milk consumption. The associations with respect to other dairy products were not significant. Adjustment for potential confounding factors, i.e. smoking, body mass index, number of childbirths, occupation and geographic area, resulted in only a minor change in the milk intake-breast cancer relation. Nor did adjustment for intake of other foodstuffs and nutrients, e.g. energy, carbohydrates, protein, fat, vitamins and trace elements, alter the results. No significant interactions between milk intake and demographic or dietary variables or time of cancer diagnosis were observed. Our data suggest that there is a protective effect, dietary or habitual, associated with consumption of milk that overwhelms the associations between different other factors and risk of breast cancer.

**Keywords:** breast; dairy product; diet; follow-up; neoplasm

The importance of dietary factors in breast cancer development has been suggested by animal studies and correlation studies between countries and in migrant populations (Rohan and Bain, 1987). A high intake of fat in particular has been suspected of being a risk factor for breast cancer. In a comparison of different countries, a higher intake of milk products, representing an important fat source in developed countries, was associated with increased breast cancer mortality (Armstrong and Doll, 1975). Analytical epidemiological studies in individuals have, however, provided less consistent results. Both the cohort and case-control studies on fat (Hunter and Willett, 1994) or milk intake (Boyd *et al.*, 1993) and breast cancer risk have been conflicting. In a small Finnish prospective study on dietary fat and breast cancer incidence in initially 20-69 years old women we, unexpectedly, found an inverse association between milk intake and breast cancer (Knekt *et al.*, 1990). Since milk intake in Finland has been among the highest in the world during the past decade we decided to investigate the associations between intake of different dietary products and breast cancer risk in greater detail during a longer follow-up period.

### Population and methods

The Mobile Health Clinic of the Social Insurance Institution carried out multiphasic screening examinations in different areas of Finland during 1966-72 (Knekt, 1988). Food consumption data were obtained from 4697 women aged 15-90 years old (mean = 39 years and s.d. = 16) and free from cancer. A modified dietary history interview method was used to survey the total habitual diet of examinees during the past year (Järvinen *et al.*, 1993). A structured interview guided by a preformed questionnaire was performed by trained interviewers. The amounts of food items consumed were assessed per day, week, month and year. Food models were used to facilitate estimation of the size of portions consumed. The amount of each individual food item per day was calculated by combining the amount of food directly reported in the interview with that derived from mixed dishes. The nutrient intakes of food items were computed using a food composition database compiled at the Social Insurance Institution. Engery intake was calculated from the amounts of protein, fat and available carbohydrates consumed.

The participants also completed a premailed questionnaire yielding information on residence, occupation, parity and smoking. Occupations were divided into main classes according to the Nordic Standard Classification of Occupations, an adaption of the ILO classification (Brockington, 1967). The women were classified according to smoking status as non-smokers (never-smokers and ex-smokers combined) and current smokers. Body height and weight were measured, and the body mass index (weight/height$^2$; kg m$^{-2}$) was calculated. Short-term and long-term repeatability of the daily consumption of milk products was estimated by repeating the dietary interviews 4-8 months and 4-7 years after the initial interviews. The intraclass correlation coefficients were 0.68 and 0.54 respectively (Järvinen *et al.*, 1993).

Information concerning subsequent cancer incidence, available through the nationwide Finnish Cancer Registry (Teppo *et al.*, 1980) was linked to the data. During a 25 year follow-up period 88 breast cancers were diagnosed. Information about mortality was based on death certificates obtained for all the deceased from the Central Statistical Office of Finland (Reunanen *et al.*, 1983).

The age-adjusted mean daily intake of different foodstuffs at different levels of milk consumption and mean daily intake of milk products among women with subsequent breast cancer and others were estimated using the general linear model (Cohen and Cohen, 1975). Cox's proportional hazards model was used to estimate the adjusted association between the intake of dairy products and risk of breast cancer (Kalbfleisch and Prentice, 1980). Potential confounding factors, e.g. energy intake, were adjusted for by including them in the model. Relative risks were computed for tertiles of dairy product consumption, using the lowest tertile as a reference category. Statistical significances were tested using the likelihood ratio test based on the Cox model.

Correspondence: P Knekt
Received 1 February 1995; revised 21 September 1995; accepted 29 September 1995

**Results**

The risk of breast cancer was highest among women over 50 years of age, in white-collar professions, with no childbirths, lean and unmarried (Table I). The mean intake of total dairy products was lower among breast cancer cases than among non-cases owing to differences in milk consumption (Table II). There was a significant inverse gradient ($P<0.01$) between the age-adjusted intake of milk and subsequent occurrence of breast cancer. The relative risk was 0.42 (95% confidence interval (CI) = 0.23–0.78) between the highest and lowest tertiles of intake (Table III); the associations for other dairy products were non-significant. Exclusion of the breast cancer cases occurring during the first five years of follow-up did not notably change the results: the relative risk of breast cancer between the highest and lowest tertiles of milk intake was 0.49 (CI = 0.28–0.87).

Milk intake depended significantly on occupation and geographic area and was higher in agriculture and in the western part of the country than elsewhere. Other potential confounding factors (i.e. region type, body mass index, parity and smoking) were, however, only weakly associated with milk intake (data not shown). Adjustment for non-dietary factors (i.e. age, smoking, body mass index, number of childbirths, occupation and geographic area) did not notably alter the association between milk intake and breast cancer; the relative risk was 0.49 (CI = 0.27–0.86) between the lowest and highest tertiles of intake. The relative risk of breast cancer between high and low levels of milk intake was also studied in strata of these factors, and no notable interactions were observed (data not shown).

There was a strong association between intake of milk and intake of energy and different foodstuffs (Table IV). Adjustment for these dietary variables, however, altered

**Table I** Age-adjusted relative risk of breast cancer in classes of potential confounding and effect modifying factors

| *Factor* | *No. of cases* | *No. at risk* | *Relative risk* | *95% confidence interval* |
|---|---|---|---|---|
| Age | | | | |
| 15–29 | 11 | 1 574 | 1.0 | |
| 30–39 | 21 | 907 | 3.37 | 1.63–6.99 |
| 40–49 | 15 | 861 | 2.71 | 1.24–5.90 |
| 50–59 | 24 | 718 | 5.98 | 2.93–12.22 |
| 60–69 | 15 | 487 | 7.33 | 3.36–16.03 |
| 70–99 | 2 | 150 | 5.82 | 1.28–26.51 |
| Occupational group | | | | |
| Agriculture | 12 | 939 | 1.0 | |
| Industry | 13 | 607 | 2.36 | 1.07–5.20 |
| Services | 28 | 1 677 | 2.16 | 1.09–4.30 |
| White-collar | 23 | 783 | 3.40 | 1.67–6.91 |
| Housewives | 11 | 686 | 1.37 | 0.60–3.10 |
| Geographic area | | | | |
| South-west | 31 | 1 147 | 1.0 | |
| South | 8 | 488 | 0.66 | 0.30–1.45 |
| Central | 13 | 612 | 0.83 | 0.43–1.58 |
| West | 3 | 412 | 0.31 | 0.09–1.02 |
| East | 15 | 1 000 | 0.69 | 0.37–1.30 |
| North | 18 | 1 038 | 0.82 | 0.45–1.49 |
| Type of region | | | | |
| Semiurban | 51 | 2 616 | 1.0 | |
| Rural | 30 | 1 726 | 0.81 | 0.51–1.27 |
| Industrial | 7 | 355 | 1.11 | 0.50–2.44 |
| Smoking | | | | |
| Non | 74 | 3 849 | 1.0 | |
| Current | 13 | 843 | 1.17 | 0.64–2.15 |
| Childbirths | | | | |
| No | 23 | 1 544 | 1.0 | |
| Yes | 64 | 3 148 | 0.89 | 0.55–1.46 |
| Body mass index, quintile | | | | |
| 0–20.94 | 13 | 915 | 1.0 | |
| 20.95–23.22 | 18 | 960 | 0.92 | 0.45–1.90 |
| 23.23–25.45 | 16 | 933 | 0.62 | 0.29–1.34 |
| 25.46–28.67 | 22 | 948 | 0.67 | 0.32–1.42 |
| 28.68–99.99 | 18 | 936 | 0.47 | 0.21–1.04 |
| Marital status | | | | |
| Unmarried | 22 | 1 305 | 1.0 | |
| Married | 50 | 2 843 | 0.64 | 0.38–1.07 |
| Divorced, Widowed | 15 | 544 | 0.68 | 0.33–1.42 |

**Table II** Mean level[a] of intake of different dairy products among breast cancer cases and non-cases

| *Dairy product (g day$^{-1}$)* | *Cases (n = 88)* *Mean* | *s.d.* | *Non-cases (n = 4 609)* *Mean* | *s.d.* |
|---|---|---|---|---|
| All | 658 | 318 | 743 | 342 |
| Milk | 432 | 313 | 531 | 319 |
| Fermented milk | 157 | 226 | 141 | 191 |
| Butter | 34 | 22 | 37 | 22 |
| Cream | 19 | 39 | 16 | 30 |
| Ice-cream | 4 | 7 | 4 | 9 |
| Cheese | 13 | 13 | 12 | 16 |

[a]Age-adjusted.

**Table III** Relative risk[a] of breast cancer between tertiles of intake of different dairy products

| *Dairy product* | *Lowest* | *Relative risk by tertile (95% confidence interval)* *Middle* | *Highest* | P *value for trend* |
|---|---|---|---|---|
| All | 1.0 | 1.11(0.70–1.75) | 0.42(0.23–0.78) | 0.02 |
| Milk | 1.0 | 0.67(0.42–1.08) | 0.42(0.24–0.74) | 0.003 |
| Fermented milk | 1.0 | 1.42(0.83–2.42) | 1.37(0.80–2.37) | 0.47 |
| Butter | 1.0 | 0.70(0.42–1.14) | 0.59(0.35–0.99) | 0.17 |
| Cream | 1.0 | 0.59(0.32–1.09) | 0.84(0.53–1.34) | 0.67 |
| Ice-cream | 1.0 | 0.95(0.56–1.60) | 0.63(0.35–1.15) | 0.32 |
| Cheese | 1.0 | 1.19(0.71–2.00) | 1.25(0.75–2.08) | 0.66 |

[a]Age-adjusted

**Table IV** Age-adjusted mean daily intake of different foodstuffs in tertiles of milk intake

| *Foodstuff* | *Lowest* | *Tertile[a] of milk intake* *Middle* | *Highest* | P-*value for trend* |
|---|---|---|---|---|
| Cereals (g) | 204 | 228 | 271 | <0.001 |
| Potatoes (g) | 149 | 169 | 201 | <0.001 |
| Vegetables (g) | 131 | 116 | 116 | <0.001 |
| Fruits and berries (g) | 164 | 141 | 133 | <0.001 |
| Margarine (g) | 6.70 | 6.50 | 6.95 | 0.32 |
| Dairy products (g) | 486 | 679 | 1060 | <0.001 |
| Meat and meat products (g) | 109 | 110 | 122 | <0.001 |
| Fish (g) | 23 | 22 | 25 | <0.001 |
| Eggs (g) | 29 | 30 | 34 | <0.001 |
| Energy (kcal) | 1789 | 2065 | 2588 | <0.001 |

[a]Tertiles of milk intake (g $day^{-1}$): <370, 370–619, ≥620.

only slightly the milk intake–breast cancer relationship; the relative risk was 0.57 (CI = 0.28–1.13) between the highest and lowest tertiles of milk intake. No significant interactions were noted.

Milk intake was significantly positively correlated with the dietary intake of carbohydrates, protein and fat; the age-adjusted partial correlation coefficients were 0.41, 0.53 and 0.55, respectively. Adjustment for these nutrients, however, did not notably alter the association between breast cancer and milk intake; the relative risk was 0.40 (CI = 0.21–0.76). Study of the association between risk of breast cancer and different nutrients (vitamins, trace elements and fatty acids) to which milk made a noticeable contribution revealed only two significant relationships: lactose with a relative risk of 0.53 (CI-0.30–0.94), and calcium with a relative risk of 0.44 (CI = 0.24–0.80). The total amount of milk fat derived from dairy products was non-significantly inversely associated with breast cancer, with a relative risk of 0.64 (CI = 0.39–1.06). Milk, lactose and calcium intake could not be included in the same model because of their high correlation. Adjustment for calcium and milk fat intake did not materially alter the association between milk and breast cancer occurrence.

### Discussion

The main finding of this longitudinal study was that milk consumption was inversely associated with breast cancer occurrence. The result is at variance with findings from intercountry comparisons associating high consumption of dairy products with breast cancer mortality (Armstrong and Doll, 1975). Few prospective studies thus far published on intake of milk or milk products combined and breast cancer incidence have revealed a significant inverse association (Toniolo *et al.*, 1994) or no association (Mills *et al.*, 1988; Ursin *et al.*, 1990). Case–control studies have resulted in inconsistent findings suggesting an inverse (Pryor *et al.*, 1989; Simard *et al.*, 1990; Kato *et al.*, 1992; Levi *et al.*, 1993), no (Lubin *et al.*, 1981; Katsouyanni *et al.*, 1986; Hirohata *et al.*, 1987; La Vecchia *et al.*, 1987; Iscovich *et al.*, 1989; van't Veer *et al.*, 1989; Ingram *et al.*, 1991; Richardson *et al.*, 1991) or a positive association (Phillips, 1975; Talamini *et al.*, 1984; Hislop *et al.*, 1986; Le *et al.*, 1986; Toniolo *et al.*, 1989; Ewertz and Gill, 1990; Mettlin *et al.*, 1990).

The discrepant results may be as a result of methodological factors. Potential bias of dietary data arising from case status may affect the results of case–control studies (Giovannucci *et al.*, 1993). Prospective studies such as this one, however, avoid this kind of error. Differences in dietary assessment methods used and their inadequacy in revealing real habitual food consumption of individuals is a major problem in all nutritional epidemiological research. The importance of milk products in the total diet is also known to vary among populations (Cramer *et al.*, 1994); thus the discrepancies between studies may also be partly due to differences in adequacy in revealing the true milk consumption in different populations. In the present study population milk was one of the staple foods, but with a wide range of variation in consumption. Any effect of milk intake on breast cancer occurrence would thus most probably be noticed in a population such as the one reported here.

It is possible that other dietary factors associated with milk consumption may afford protection against breast cancer. Several studies have used rather limited questionnaires on food consumption (Mills *et al.*, 1988; Ewertz and Gill, 1990; Hislop *et al.*, 1986; Mettlin *et al.*, 1990; Ursin *et al.*, 1990; Kato *et al.*, 1992), thus restricting the opportunities to adjust for potential confounding factors due to other dietary components. We applied a survey method intended to reveal the total dietary intake, enabling us to study potential dietary confounders; adjustment for such did not, however, notably alter the associations. Although we found that the association between milk consumption and breast cancer was independent of several dietary factors, the possibility cannot be excluded that differences in milk intake reflect factors or food consumption patterns more specifically associated with breast cancer development.

The discrepant results between different studies may in part be due to the introduction of a possible confounding effect as a result of unsatisfactory control for health, behavioural and environmental factors. Alcohol consumption is a potential confounder (Longnecker *et al.*, 1988) not measured in the present study. The effect of alcohol was, however, probably negligible here as drinking was very uncommon among Finnish women at the time of the baseline study (Simpura, 1987). Reproductive factors (Kelsey and Whittemore, 1994) may have confounded the results as only the number of births was available. Adjustment for several potential confounding factors, including number of births, did not notably alter the associations, however.

Milk comprises a complex mixture of major and minor nutrients. The association observed may be caused by a protective effect of some or a combination of these. Of single nutrients we found lactose and calcium to be significantly inversely associated with breast cancer occurrence. Because the intakes of these variables were strongly associated with milk intake no firm conclusions can be drawn, however.

Lactose intake was mainly determined by milk and fermented milk consumption. In some previous studies use of fermented milk (Le *et al.*, 1986; van't Veer *et al.*, 1989) was found to be inversely associated with breast cancer risk, possibly due to the favourable effects of lactic acid bacteria appearing in these products. A similar association was found for cheese intake in one study (van't Veer *et al.*, 1989). The majority of previous studies, however, reported a positive association (Le *et al.*, 1986; Toniolo *et al.*, 1989; Levi *et al.*, 1993) or no association (Phillips, 1975; Lubin *et al.*, 1981; Hirohata *et al.*, 1987; Mills *et al.*, 1988; Iscovich *et al.*, 1989; Richardson *et al.*, 1991) between cheese consumption and breast cancer risk. In the present study no beneficial effects were ascribed to the use of fermented milk products.

An adverse effect of hydrolysed milk sugar, galactose, on ovarian function and fertility has been suggested (Cramer *et al.*, 1994). Since breast cancer is dependent on hormonal factors, potential inhibition of hormonal function by lactose intake might offer one explanation for the suggested effect of milk intake. Such an effect would be most probable in the type of population under study here, in which the ability to digest lactose is sustained beyond childhood in the majority of the population (Cramer *et al.*, 1994).

High calcium intake was associated with a lower breast cancer incidence in the present study. Similar findings have been reported with respect to colon cancer (Sorenson *et al.*, 1988). They may possibly be due to the fact that calcium ions from the diet may provide protection by binding fatty acids and bile acids in insoluble compounds (Newmark *et al.*, 1984). A few previous studies (Katsouyanni *et al.*, 1988) have reported no favourable effect of dietary calcium on breast cancer occurrence. As adjustment for calcium intake in our study did not eliminate the association between milk intake and breast cancer occurrence, calcium is apparently not solely responsible for the observed effect of milk.

Here, milk fat accounted for a major part of total dietary fat intake. In comparison with other foods milk fat contains exceptionally high amounts of saturated fat. Studies have suggested, though not consistently, that a high intake of saturated fat may be associated with an elevated risk of breast cancer (Boyd *et al.*, 1993; Hunter *et al.*, 1994). There was no significant association between total or saturated fat intake and breast cancer incidence in the present population (Knekt *et al.*, 1990). Like several other researchers (Le *et al.*, 1986; Toniolo *et al.*, 1989; Ewertz and Gill, 1990; Ingram *et al.*, 1991; Richardson *et al.*, 1991), we, too, found no association between butter intake and breast cancer incidence. The intake of total milk fat was, however, inversely related to breast cancer risk. It may be noted that milk fat is a good source of conjugated linoleic acid isomer (Chin *et al.*, 1992), which has been shown to be a very efficient suppressor of mammary tumours in animal experiments (Ip *et al.*, 1991).

There are several other compounds in milk that may possibly be involved in protection against breast cancer. Despite numerous studies on antioxidant vitamin status and breast cancer risk, current data do not support the hypothesis of a protective effect of these compounds against the disease (Garland *et al.*, 1993). In keeping with these findings, adjustment for antioxidants did not notably alter the association between milk intake and breast cancer incidence in our study. Analyses of several other vitamins and trace elements gave the same negative result.

In summary, we found an inverse relation between dietary intake of milk and subsequent incidence of breast cancer. Despite the study of different compounds of milk, this inverse association remains an enigma. The association persisted after control for dietary and life-style factors associated with milk consumption. The possible effects of confounding can still not be excluded, however. Further cohort studies based on large populations with high milk intake should thus focus on this topic.

**Acknowledgements**
This study was supported by the Swedish Cancer Foundation.

### References

ARMSTRONG B AND DOLL P. (1975). Environmental factors and cancer incidence and mortality in different countries, with special reference to dietary practices. *Int. J. Cancer*, **15**, 617–631.

BOYD NF, MARTIN LJ, NOFFEL M, LOCKWOOD GA AND TRITCHLER DL. (1993). A meta-analysis of studies of dietary fat and breast cancer risk. *Br. J Cancer*, **68**, 627–636.

BROCKINGTON F. (1967). *World Health, Appendix VIII, The International Standard Classification of Occupations*, 2nd edn. pp. 331–339. Churchill: London.

CHIN SF, LIU W, STORKSON JM, HA YL AND PARIZA MW. (1992). Dietary sources of conjugated dienoic isomers of linoleic acid, a newly recognized class of anticarcinogens. *J. Food Compos. Anal.*, **5**, 185–197.

COHEN J AND COHEN P. (1975). *Applied Multiple Regression/ Correlation Analysis for the Behavioral Sciences*. Wiley: New York.

CRAMER DW, XU H AND SAHI T. (1994). Adult hypolactasia, milk consumption, and age-specific fertility. *Am. J. Epidemiol.*, **139**, 282–289.

EWERTZ M AND GILL C. (1990). Dietary factors and breast-cancer risk in Denmark. *Int. J. Cancer*, **46**, 779–784.

GARLAND M, WILLETT WC, MANSON JAE AND HUNTER DJ. (1993). Antioxidant micronutrients and breast cancer. *J. Am. Coll. Nutr.*, **12**, 400–411.

GIOVANNUCCI E, STAMPFER MJ, COLDITZ GA, MANSON JAE, ROSNER BA, LONGNECKER M, SPEIZER FE AND, WILLETT WC. (1993). A comparison of prospective and retrospective assessments of diet in the study of breast cancer. *Am. J. Epidemiol.*, **137**, 502–511.

HIROHATA T, NOMURA AMY, HANKIN JH, KOLONEL LN AND LEE J. (1987). An epidemiologic study on the association between diet and breast cancer. *J. Natl Cancer Inst.*, **78**, 595–600.

HISLOP TG, COLDMAN AJ, ELWOOD JM, BRAUER G AND KAN L. (1986). Childhood and recent eating patterns and risk of breast cancer. *Cancer Detect. Prev.*, **9**, 47–58.

HUNTER DJ AND WILLETT WC. (1994). Diet, body build, and breast cancer. *Annu. Rev. Nutr.*, **14**, 393–418.

INGRAM DM, NOTTAGE E AND ROBERTS T. (1991). The role of diet in the development of breast cancer: a case–control study of patients with breast cancer, benign epithelial hyperplasia and fibrocystic disease of the breast. *Br. J. Cancer*, **64**, 187–191.

IP C, CHIN SF, SCIMECA JA AND PARIZA MW. (1991). Mammary cancer prevention by conjugated dienoic derivative of linoleic acid. *Cancer Res.*, **51**, 6118–6124.

ISCOVICH JM, ISCOVICH RB, HOWE G, SHIBOSKI S AND KALDOR JM. (1989). A case–control study of diet and breast cancer in Argentina. *Int. J. Cancer*, **44**, 770–776.

JÄRVINEN R, SEPPÄNEN R AND KNEKT P. (1993). Short-term and long-term reproducibility of dietary history interview data. *Int. J. Epidemiol.*, **22**, 520–527.

KALBFLEISCH JD AND PRENTICE RL. (1980). *The Statistical Analysis of Failure Time Data*. Wiley: New York.

KATO I, MIURA S, KASUMI F, IWASE T, TASHIRO H, FUJITA Y, KOYAMA H, IKEDA T, FUJIWARA K, SAOTOME K, ASAISHI K, ABE R, NIHEI M, ISHIDA T, YOKOE T, YAMAMOTO H AND MURATA M. (1992). A case-control study of breast cancer among Japanese women: with special reference to family history and reproductive and dietary factors. *Breast Cancer Res. Treat.*, **24,** 51-59.

KATSOUYANNI K, TRICHOPOULOS D, BOYLE P, XIROUCHAKI E, TRICHOPOULOU A, LISSEOS B, VASILAROS S AND MACMAHON B. (1986). Diet and breast cancer: a case-control study in Greece. *Int. J. Cancer*, **38,** 815-820.

KELSEY JL AND WHITTEMORE AS. (1994). Epidemiology and primary prevention of the breast, endometrium and ovary. A brief overview. *Ann. Epidermal*, **4,** 89-95.

KNEKT P. (1988). *Serum Alpha-tocopherol and the Risk of Cancer*, Series ML: 83. The Social Insurance Institution: Finland.

KNEKT P, ALBANES D, SEPPÄNEN R, AROMAA A, JÄRVINEN R, HYVÖNEN L, TEPPO L AND PUKKALA E. (1990). Dietary fat and risk of breast cancer. *Am. J. Clin. Nutr.*, **52,** 903-908.

LA VECCHIA C, DECARLI A, FRANCESCHI S, GENTILE A, NEGRI E AND PARAZZINI F. (1987). Dietary factors and the risk of breast cancer. *Nutr. Cancer*, **10,** 205-214.

LE MG, MOULTON LH, HILL C AND KRAMAR A. (1986). Consumption of dairy produce and alcohol in a case-control study of breast cancer. *J. Natl Cancer Inst.*, **77,** 633-636.

LEVI F, LA VECCHIA C, GULIE C AND NEGRI E. (1993). Dietary factors and breast cancer risk in Vaud, Switzerland. *Nutr. Cancer*, **19,** 327-335.

LONGNECKER MP, BERLIN JA, ORZA MJ AND CHALMERS TC. (1988). A meta-analysis of alcohol consumption in relation to risk of breast cancer. *JAMA*, **260,** 652-656.

LUBIN JH, BURNS PE, BLOT WJ, ZIEGLER RG, LEES AW AND FRAUMENI JF Jr. (1981). Dietary factors and breast cancer risk. *Int. J. Cancer*, **28,** 685-689.

METTLIN CJ, SCHOENFELD ER AND NATARAJAN N. (1990). Patterns of milk consumption and risk of cancer. *Nutr. Cancer*, **13,** 89-99.

MILLS PK, ANNEGERS JF AND PHILLIPS RL. (1988). Animal product consumption and subsequent fatal breast cancer risk among Seventh-day Adventists. *Am. J. Epidemiol.*, **127,** 440-453.

NEWMARK HL, WARGOVICH MJ AND BRUCE WR. (1984). Colon cancer and dietary fat, phosphate, and calcium: a hypothesis. *J. Natl Cancer Inst.*, **72,** 1323-1325.

PHILLIPS RL. (1975). Role of life-style and dietary habits in risk of cancer among Seventh-Day Adventists. *Cancer Res.*, **35,** 3513-3522.

PRYOR M, SLATTERY ML, ROBISON LM AND EGGER M. (1989). Adolescent diet and breast cancer in Utah. *Cancer Res.*, **49,** 2161-2167.

REUNANEN A, AROMAA A, PYÖRÄLÄ K, PUNSAR S, MAATELA J AND KNEKT P. (1983). The Social Insurance Institution's Coronary Heart Disease Study. Baseline data and 5-year mortality experience. *Acta Med. Scand.*, **673,** (suppl.) 12.

RICHARDSON S, GERBER M AND CENEE S. (1991). The role of fat, animal protein and some vitamin consumption in breast cancer: a case control study in southern France. *Int. J. Cancer*, **48,** 1-9.

ROHAN TE AND BAIN CJ. (1987). Diet in the etiology of breast cancer. *Epidemiol. Rev.*, **9,** 120-145.

SIMARD A, VOBECKY J AND VOBECKY JS. (1990). Nutrition and life-style factors in fibrocystic disease and cancer of the breast. *Cancer Detect. Prev.*, **14,** 567-572.

SIMPURA J. (ed). (1987). *Finnish Drinking Habits. Results from Interview Studies Held in 1968, 1976, and 1984*. The Finnish Foundation for Alcohol Studies, 35, pp. 272. Gummerus: Jyväskylä.

SORENSON AW, SLATTERY ML AND FORD MH. (1988). Calcium and colon cancer: a review. *Nutr. Cancer*, **11,** 135-145.

TALAMINI R, LA VECCHIA C, DECARLI A, FRANCESCHI S, GRATTONI E, GRIGOLETTO E, LIBERATI A AND TOGNONI G. (1984). Social factors, diet and breast cancer in a northern Italian population. *Br. J. Cancer*, **49,** 723-729.

TEPPO L, PUKKALA E, HAKAMA M, HAKULINEN T, HERVA A AND SAXÉN E. (1980). Way of life and cancer incidence in Finland. A municipality-based ecological analysis. *Scand. J. Soc. Med.*, (suppl.) 19.

TONIOLO P, RIBOLI E, PROTTA F, CHARREL M AND CAPPA APM. (1989). Calorie-providing nutrients and risk of breast cancer. *J. Natl Cancer Inst.*, **81,** 278-286.

TONIOLO P, RIBOLI E, SHORE RE AND PASTERNACK BS. (1994). Consumption of meat, animal products, protein, and fat and risk of breast cancer: a prospective cohort study in New York. *Epidemiology*, **5,** 391-397.

URSIN G, BJELKE E, HEUCH I AND VOLLSET SE. (1990). Milk consumption and cancer incidence: a Norwegian prospective study. *Br. J. Cancer*, **61,** 454-459.

VAN'T VEER P, DEKKER JM, LAMERS JWJ, KOK FJ, SCHOUTEN EG, BRANTS HAM, STURMANS F AND HERMUS RJJ. (1989). Consumption of fermented milk products and breast cancer: a case-control study in the Netherlands. *Cancer Res.*, **49,** 4020-4023.